# Mechanism for Tuning the Hydrophobicity of Microfibrillated Cellulose Films by Controlled Thermal Release of Encapsulated Wax

**DOI:** 10.3390/ma7117196

**Published:** 2014-10-28

**Authors:** Vibhore Kumar Rastogi, Dirk Stanssens, Pieter Samyn

**Affiliations:** 1 Chair for Bio-based Materials Engineering, Faculty for Environment and Natural Resources, Freiburg Research Institute for Advanced Studies (FRIAS), University of Freiburg, Werthmannstrasse 6, 79085 Freiburg, Germany; E-Mail: vibhore.rastogi@fowabi.uni-freiburg.de; 2 Hermann Staudinger Graduate School, University of Freiburg, Hebelstrasse 27, 79104 Freiburg, Germany; 3 Topchim N.V., Nijverheidsstraat 98, 2160 Wommelgem, Belgium; E-Mail: dirk.stanssens@topchim.be

**Keywords:** microfibrillated cellulose, hydrophobicity, encapsulation, wax, release

## Abstract

Although films of microfibrillated cellulose (MFC) have good oxygen barrier properties due to its fine network structure, properties strongly deteriorate after absorption of water. In this work, a new approach has been followed for actively tuning the water resistance of a MFC fiber network by the inclusion of dispersed organic nanoparticles with encapsulated plant wax. The modified pulp suspensions have been casted into films and were subsequently cured at 40 to 220 °C. As such, static water contact angles can be specifically tuned from 120 to 150° by selection of the curing temperature in relation with the intrinsic transition temperatures of the modified pulp, as determined by thermal analysis. The appearance of encapsulated wax after curing was followed by a combination of morphological analysis, infrared spectroscopy and Raman mapping, showing balanced mechanisms of progressive release and migration of wax into the fiber network controlling the surface properties and water contact angles. Finally, the appearance of nanoparticles covered with a thin wax layer after complete thermal release provides highest hydrophobicity.

## 1. Introduction

Microfibrillated cellulose (MFCs) possesses numerous interesting properties attributed to the formation of a dense fibrous network with high surface area and aspect ratio, high stiffness and tensile strength, and good oxygen barrier properties. In parallel with its non-toxicity, biodegradability and origin from various renewable resources or secondary side-stream materials (e.g., fine paper fibers or agricultural waste), the MCF has become a suitable candidate as a natural reinforcing material for bio-composites [1] or in packaging applications, used as free-standing cast film [2] or coating additive [3]. However, the full exploitation of these properties is always masked by the strong hydrophilicity leading to high fiber-to-fiber affinity and poor adhesion or dispersion in a non-polar matrix. Deterioration of oxygen barrier properties [4] is another major problem associated with the hydrophilic nature of cellulose that makes it sensitive to water or moisture adsorption consequently leading to weakening and swelling of the cellulose network. Accordingly, major efforts are generally put forward to improve the water resistance of single cellulosic paper fibers by hydrophobic modifications [5].

Most of the cited surface modifications for cellulose follow traditional chemical routes where the hydroxyl groups are modified by “grafting-on” and “grafting-to” reactions [6,7]. With the advent of nanotechnology, horizons towards novel interface engineering starts to play a critical role [8]. Specifically for providing (super-)hydrophobic properties, nano-engineering approaches are applied for imparting the chemical surface composition and dual-scale surface roughness [9]. The hydrophobization of cellulose surfaces by nanoparticle decoration (*i.e.*, physical or chemical absorption) has been realized in our previous work with styrene maleimide nanoparticles [10]. Similarly, cellulose model surfaces were modified by adsorption of cationic polymer latexes particles [11]. The nanotechnological approaches also enable to “actively” tune the surface functionalities of papers and/or cellulose fibers after deposition of a basic coating material. As such, a novel range of intelligent responsive functional materials can be created to tune the surface functionalities of single cellulose fibers or fiber networks.

The formation of functional papers has been reported for different applications, e.g., fragrance papers [12], anti-bacterial paper [13], insect-repellant paper [14], color-producing paper [15] and photoactive papers [16]. These papers utilize the native properties of the functional materials that were initially protected from environmental factors by microencapsulation techniques, but can also be used for the controlled release of functional materials in response to external stimuli applied such as mechanical stress [12], temperature [14], light [16] and humidity [15], as such, specifically desired “user-properties” can be delivered “on-demand”. Paper coatings can benefit from the encapsulation of kaolin clay into starch, resulting in strength increase per unit of weight [17]. The encapsulated products used in paper industry mostly include inorganic fillers [18]. However, the encapsulation at nanoscale is more tedious and has been realized less frequently. Recently, a category of hybrid organic nanoparticles was synthesized by encapsulating different vegetable oils as the barrier coatings formulations to improve the hydrophobicity of the single cellulosic fibers [10] or paper as a whole [19]. The thermal curing of nanoparticle paper coatings was also seen an effective way to tune the surface hydrophobicity by simultaneous changes in surface chemistry and topography [20]. The thermal response has also been exploited for creating superhydrophobic papers with cellulose stearoyl ester [21]. Thermal curing can augment the wettability of a paper fabricated by adsorbed colloidal paraffin wax in layer-by-layer (LbL) structure by initiating cross-linking between the layers and fibers which further increases the surface roughness followed by an increase in contact angle up to 150°, probably because of intensification of wax hydrophobic properties [22].

In our previous report [23] we have developed a novel approach to hydrophobize the MFCs by the deposition of hybrid nanoparticles with encapsulated plant wax nanoparticles. Combining the aforementioned mechanisms, the current work is in the continuation of our previous work to determine the underlying mechanism responsible for systematically tuning the wettability of films with surface-modified MFC, by controlled thermal release of encapsulated plant wax that was a priori stored within the MFC film. As such, a single chemical process can be used for the hydrophobic modification of the MFC and the required water repellent properties can be “actively” tuned afterwards, upon heating as a function of temperature and time depending on the required user properties. It will be shown that the obtained water contact angles depend on the combined release and migration effects of the hydrophobic agent, leading to maximum hydrophobicity.

## 2. Results and Discussion

### 2.1. Thermal Analysis of Modified MFC Fibers

**Figure 1:**
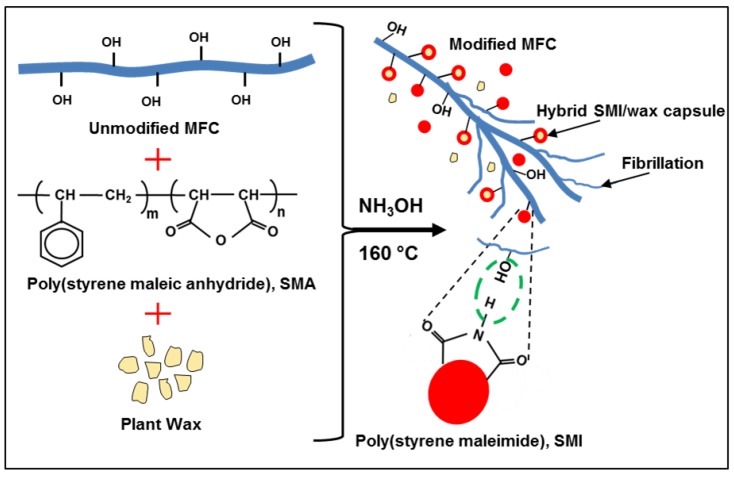
Fabrication scheme for modified microfibrillated cellulose with detailed interaction at the nanoparticles surface.

After chemical modification MFC pulp fibers, the inclusion of poly(styrene-*co*-maleimide) (SMI) and wax nanoparticles within a homogeneous dispersion of MFC + SMI/wax and the favorable deposition of nanoparticles onto the fiber surfaces was ensured (see Figure 1 for fabrication scheme) in parallel with previous preliminary evaluations [23]. In order to better understand the thermal stability and behavior of the modified MFC films, additional thermal analysis has now been done on native and modified MFC pulp fibers after freeze-drying, while pure SMI and hybrid SMI/wax nanoparticles without fibers were taken as reference materials. The thermal properties of these materials were analyzed by thermogravimetric analysis (TGA), differential scanning calorimetry (DSC) and dynamic mechanical analysis (DMA), as discussed below. The observed intrinsic transition temperatures of the modified fibers will have important influences in further controlling the thermal release of wax in the cast MFC films, as discussed in next paragraph.

#### 2.1.1. Thermogravimetric Analysis

**Figure 2:**
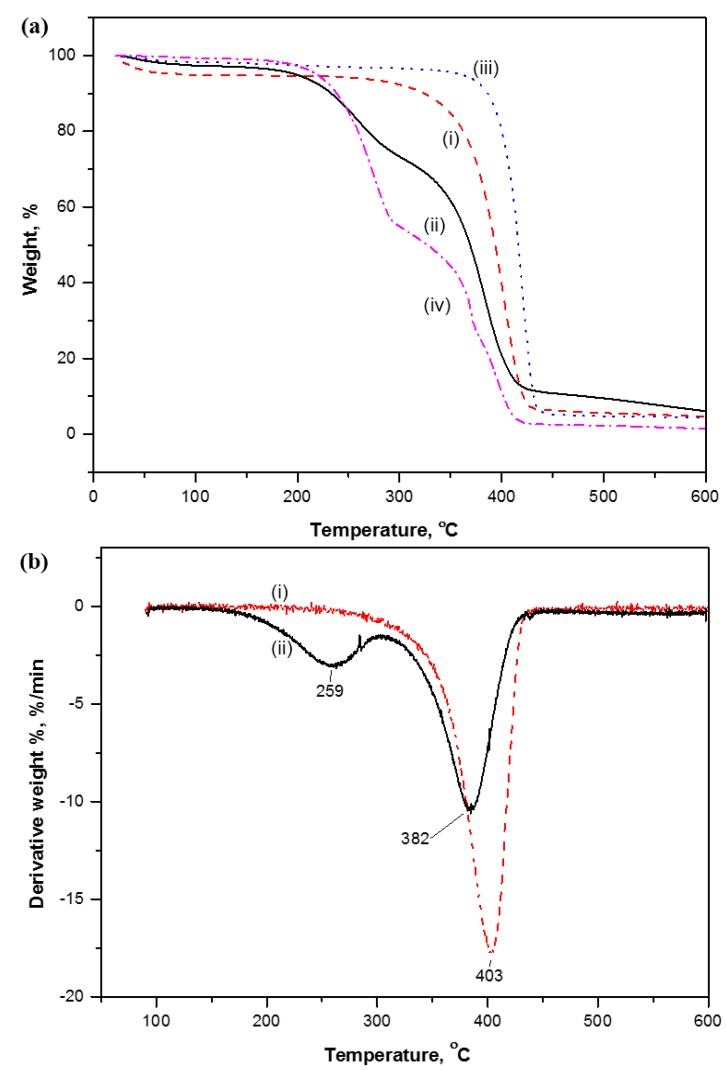
Thermogravimetric analysis representing (**a**) weight loss; (**b**) differential weight loss of (i) unmodified microfibrillated cellulose (MFC); (ii) modified MFC; (iii) pure styrene-maleimide (SMI) and (iv) SMI/wax.

The thermal degradation of the different fiber and nanoparticle materials at temperatures between 23 and 600 °C is illustrated by TGA analysis (nitrogen atmosphere) in Figure 2.

According to the weight loss curves (Figure 2a), pure SMI and unmodified MFC pulp were found to have better stability than modified MFC pulp and hybrid SMI/wax nanoparticles. Notably, the modified MFC has intermediate thermal stability in between SMI/wax and unmodified MFC, as confirming its “composite” character containing both MFC and SMI/wax components. The thermal degradation of the modified MFC occurs in two steps, representing (i) the wax degradation at temperatures of 259 °C; and (ii) fiber degradation at temperatures of 382 °C. At temperatures below 200 °C, however, full stability of modified MFC is observed, while the slight weight loss is only due to residual bound water.

As detailed in a derivative thermogravimetric analysis curve of unmodified and modified MFC (Figure 2b), the main temperature for fiber degradation of modified MFC (382 °C) is somewhat lower than for unmodified MFC (403 °C). The latter can be attributed to slightly further fibrillation of the cellulose fibers during reaction (mainly during the ammonolysis phase) [23], resulting in a higher free fiber surface area that is thermally less stable. Thermal degradation of these materials under flowing oxygen can further increase the rate of degradation, with a third degradation peak appearing in the latter stages (see Supplementary information S1).

#### 2.1.2. Differential Scanning Calorimetry

**Figure 3:**
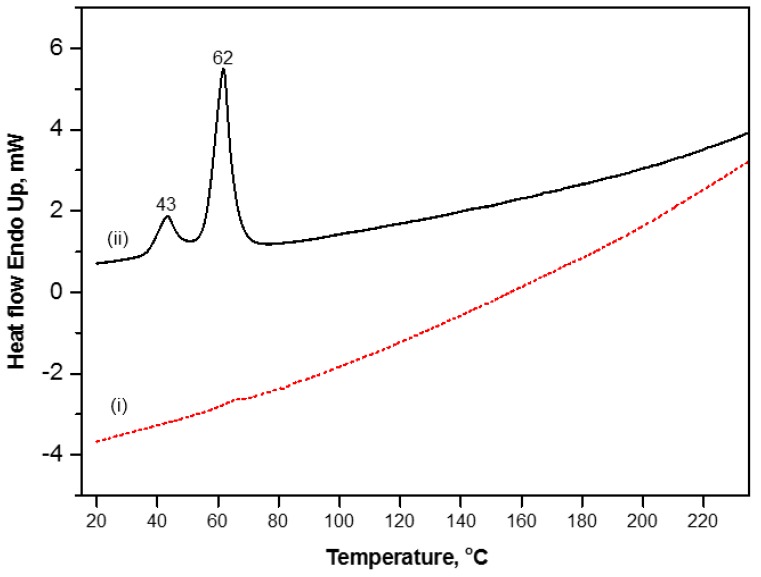
Differential Scanning Calorimetry of (i) unmodified MFC and (ii) modified MFC, curves are shifted along Y-axis to have the better visibility.

The DSC heat flow curve in Figure 3, is used to detect the phase transitions in the modified and unmodified MFC in the temperature region of 0 to 250 °C. This temperature region is specifically detailed as it will be of interest for further thermal release studies. The pure MFC did not show any first-order phase transition over the whole temperature range, possibly due to the restriction in the free molecular motion of the cellulose main chain by inter-molecular hydrogen bonding that is heavily expressed in the fine fibrous network [24]. Otherwise, two melting peaks were observed for modified MFC in low temperature regime at 43 and 62 °C, possibly attributed to the presence of some “free wax” in the system. The efficiency of the reaction is confirmed by the total amount of added wax detected in TGA (25 wt%), while an estimated amount of 20%–25% total wax remains present in the fiber network as non-bound to SMI. Both melting temperatures are characteristic for short and long hydrocarbon alkane chains of the wax, respectively. However, clear glass transition temperatures (*T_g_*) of SMI/wax nanoparticles present inside the modified MFC were not detected. In parallel, it is known that pure SMI nanoparticles have a glass transition temperature of *T_g_* = 180 °C as determined by DSC [20], while also no clear glass transition temperatures were detected for similar hybrid SMI/oil nanoparticles [19]. The glass transition temperatures for different SMI/oil nanoparticles (e.g., soy-oil, corn-oil, rapeseed-oil, palm-oil) could only be detected in previous work by temperature modulated DSC measurements, and is at around *T_g_* = 160 to 170 °C [25]. The latter illustrates that SMI forms a well-ordered complex structure in presence of oils. Similarly, in the present case of SMI/wax nanoparticles, the interactions between SMI and wax may induce the formation of some semi-organized structure that limits the molecular mobility (e.g., due to the presence of long alkaline chains and/or partial crystallization of the wax), and therefore has no clear glass transition temperatures. Moreover, the molecular relaxations occurring during the glass transition may be further hindered and/or retarded when the nanoparticles are trapped into the dense MFC fiber network. In this view, the DSC data is a good indication that a homogeneous distribution of SMI/wax has been obtained within the MFC pulp with eventually good interactions between the SMI/wax and the cellulose fibers.

#### 2.1.3. Dynamic Mechanical Analysis

**Figure 4:**
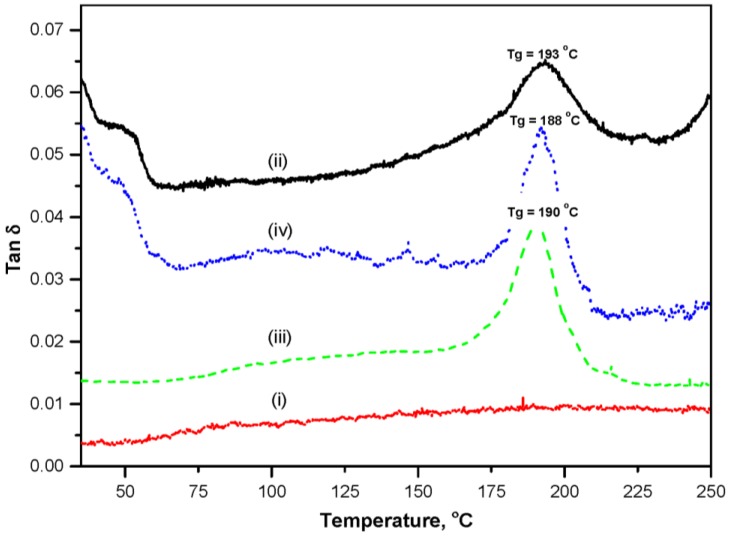
Dynamic mechanical analysis of (i) unmodified MFC; (ii) modified MFC; (iii) pure SMI and (iv) SMI/wax, curves are shifted along Y-axis to have the better visibility.

As SMI/wax and modified MFC may show complex relaxation mechanisms as outlined above, dynamic mechanical analysis was used for better visualization of the transition phenomena. In parallel with the previous visualization of glass transition temperatures for complex SMI structures by thermal modulation [20], also dynamic modulations may enhance the molecular relaxation phenomena. From the calculated loss factor (tan δ = E''/E' with E'' = loss modulus out-of-phase, and E' = storage modulus in-phase) over the temperature interval 30 to 250 °C in Figure 4, the glass transition temperatures *T_g_* of modified MFC, SMI/wax mixture and pure SMI can be very clearly determined. In parallel with DSC measurements, the native MFC pulp fibers have no transition temperature. Otherwise, the glass transition temperature of modified MFC (*T_g_* = 193 °C) is higher than for SMI (*T_g_* = 190 °C) and SMI/wax (*T_g_* = 188 °C), while also the intensity of the loss factor at the glass transition is lower for modified MFC. Both observations confirm the formation of more complex interactions when SMI/wax is trapped in the fiber network compared to the situation of SMI/wax hybrid nanoparticles. The high value of *T_g_* for modified MFC consequently leads to high stability against softening during further heat treatments. In addition, it is also possible to notice the presence of free wax in modified MFC as a broad peak ranging from 40–60 °C (in agreement with DSC). In contrast, no transitions are observed in the unmodified MFC (same as in DSC). In conclusion, a broad range for heat treatment temperatures can be applied below the glass transition temperature, without thermally degrading the integrity of the structure. Moreover, it is clear that the modified MFC network is very to dynamic forces, which make them potentially favorable to be used as active soft materials acting on external stimuli such as temperature and external loads.

### 2.2. Morphological Analysis

#### 2.2.1. Scanning Electron Microscopy

After casting the modified MFC pulp into thin films, they were thermally cured at different temperatures up to 220 °C and for different times up to 6 h. The morphology of the non-cured (room temperature) and cured MFC films was studied by scanning electron microscopy (SEM). The surface morphology varies consistently with temperatures and time, as described below, and morphological changes either reflecting the presentation of wax in combination with smoothening and/or roughening will have important consequences on the film hydrophobicity measured in next parts.

**Figure 5:**
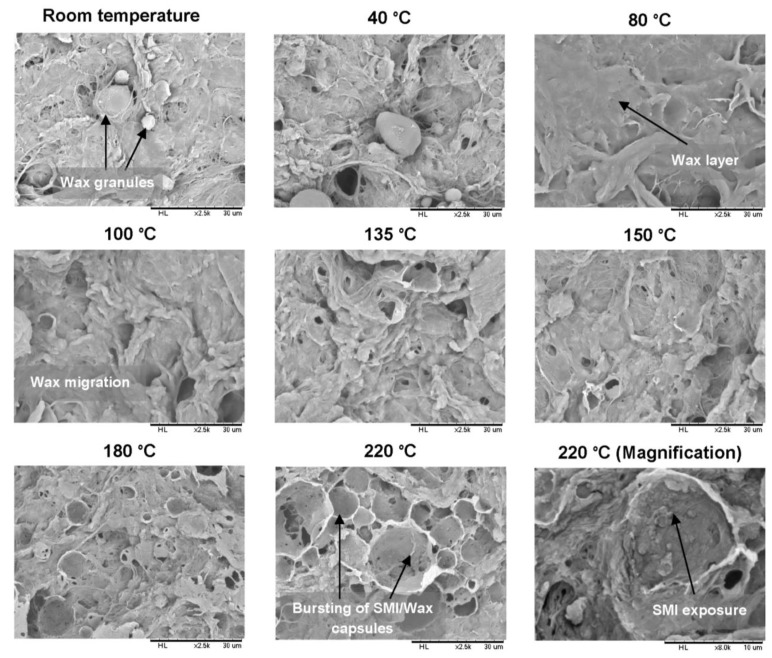
Morphology of non-cured and cured modified MFC films at different temperatures for 2 h by scanning electron microscopy (SEM).

In Figure 5, the morphology of non-cured MFC films indicates a highly heterogeneous MFC film surface having free wax granules at the surface. By increasing the temperature up to 80 °C, the surface free wax melted and spread uniformly over the surface resulting into a more continuous layer on the top of the MFC composite films, almost completely covering the fibrous structure. Further increase in temperature up to 150 °C caused the migration of the top wax layer into the bulk of the dense porous MFC film. In addition, some burst capsule-like structures also appeared at the same temperature. These structures are supposed to represent the hybrid SMI/wax capsules (further confirmed by FTIR, see later) present on the surface of fibers and incorporated in the fiber network, where wax is encapsulated by SMI and released upon after bursting. When the films were heated to 180 °C (near to *T_g_* of the hybrid SMI/wax capsules) and 220 °C (higher than the *T_g_* of the hybrid SMI/wax capsules), formation of these structures intensified, confirming the bursting of more capsules near and above their *T_g_* in parallel with the weakening of the polymeric encapsulant. This ultimately results into the complete exposure of the wax and SMI nanoparticles (Figure 5, 220 °C magnification) along with the further scope of imidization that proceeds at high curing temperatures. Consequently, the surface heterogeneity of the MFC films increased as a function of curing temperatures and times, which leads to the formation of dual scale micro-nano roughness that might enhance hydrophobicity, as explained further.

**Figure 6:**
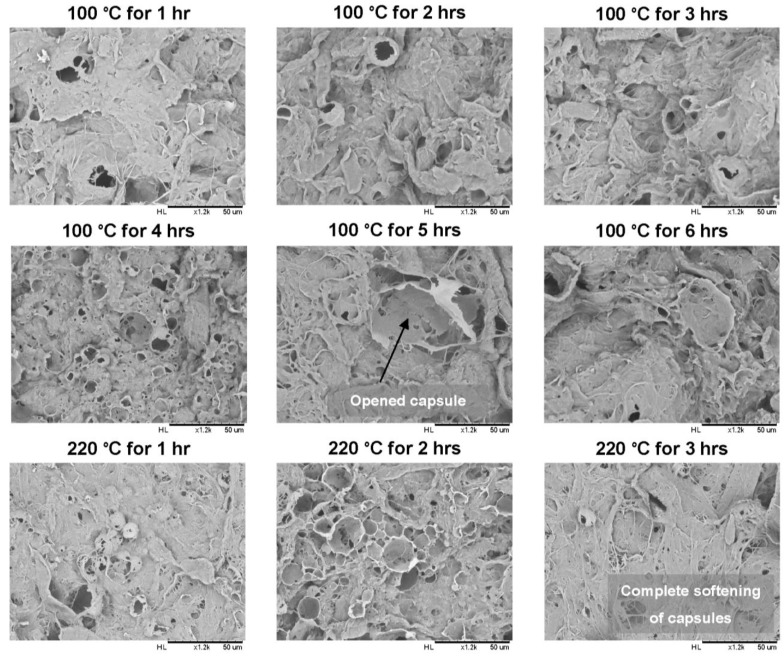
Morphology of cured modified MFC films at 100 and 220 °C for different curing times by scanning electron microscopy (SEM).

In Figure 6, the effect of increasing curing time on the modified MFC film morphology is illustrated. A same consistent trend can be observed when the films were heated at 100 and 220 °C for different times: (i) bursting capsules of SMI/wax appeared gradually and intensified over time; until (ii) complete opening and flattening of these capsules. Further details of the burst capsules (see Supplementary information S2) illustrate the complex structure of the walls and inner surface, with submicron wall thickness. In conclusion, the surface morphology gradually changes during curing due to the combined effects of progressive exposure of wax through bursting of the capsules and migration of the wax into the MFC fibrous film.

#### 2.2.2. Atomic Force Microscopy

**Figure 7:**
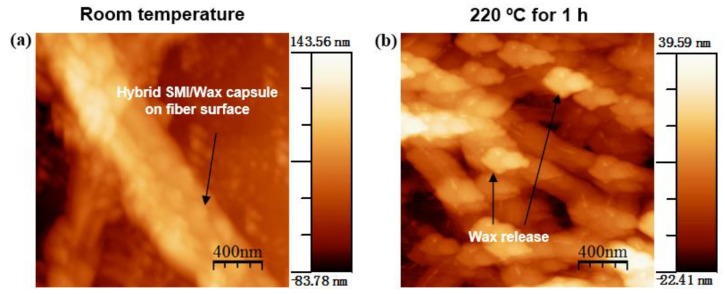
Atomic force microscopy (AFM) morphology of modified MFC films (**a**) non-cured; and (**b**) cured at maximum temperatures of 220 °C.

To understand and visualize the mechanism of wax release more clearly at nanoscale fiber-level, an AFM height image of modified MFC pulp was taken instead of using the compact MFC films (Figure 7). The originally modified pulp shows a homogeneous and relatively dense decoration of the single MFC fibers with nanoparticles (diameter around 100 nm). At higher temperatures, the wax release from hybrid SMI/wax capsules adsorbed on the modified MFC induces a different morphology and coverage of the fibers. Curing temperature (220 °C) above the glass transition temperature (193 °C) of modified MFC triggered the release of encapsulated wax by softening and bursting of hybrid SMI/wax capsules, leading to the deposition of very thin homogenous wax layer over each fiber present. The amount of the deposited wax was calculated from a line-profile over the single fiber and wax particles (see Supplementary information S3). A thin wax patch layer of thickness 15–25 nm was found to be deposited on fibers after curing at 220 °C for 1 h.

### 2.3. Chemical Analysis

#### 2.3.1. Infrared Spectroscopy

**Figure 8:**
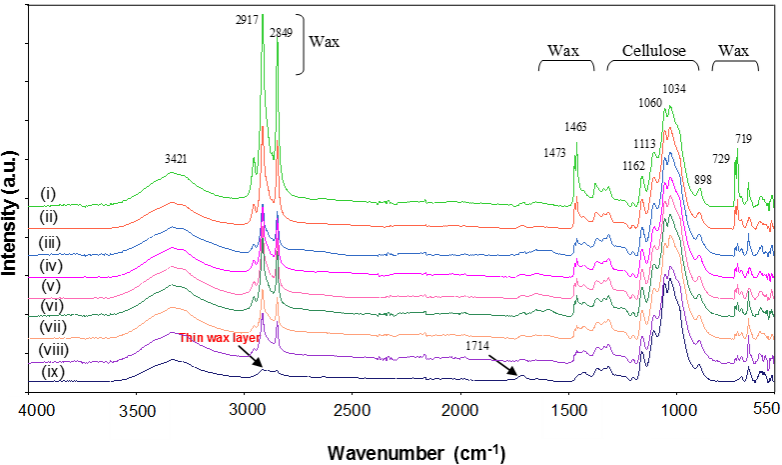
Fourier transform infrared (FTIR) spectra for (i) non-cured modified MFC films and after curing at different temperatures of (ii) 40 °C (iii) 60 °C (iv) 80 °C (v) 100 °C (vi) 135 °C (vii) 150 °C (viii) 180 °C (ix) 220 °C.

**Figure 9:**
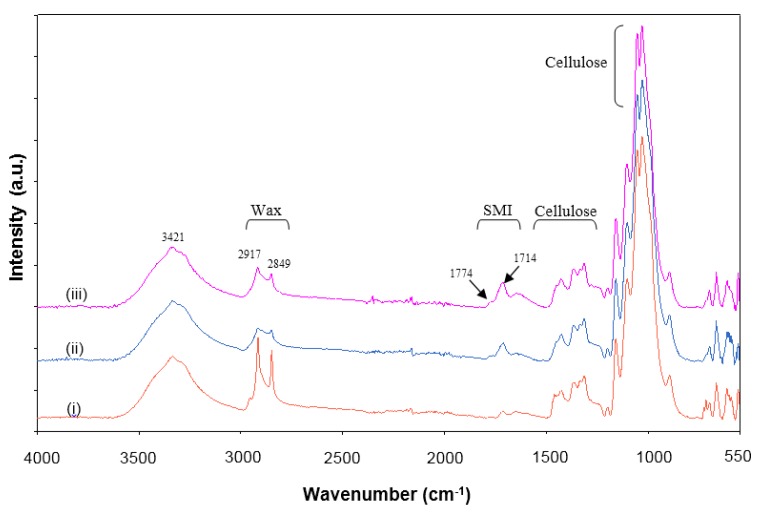
FTIR spectra for cured modified MFC films at 220 °C for different curing times of (i) 1 h; (ii) 2 h; and (iii) 3h.

The Fourier transform infrared (FTIR) spectra for non-cured and thermally cured modified MFC films after supplementary heat treatments are illustrated for different temperatures at 2 h. Characteristics absorption bands for cellulose [26] and wax [27] are prominently visible along with some minor band of SMI (1714 cm^−1^) [28], as indicated on the spectra. All spectra were baseline corrected and normalized around the cellulose C-O-C ring stretching region (1162, 1113, 1060, 1034 and 898 cm^−1^), as changes in encapsulated material after heat treatment can be expressed relatively to the cellulose structure that remains almost unaffected in the temperature region (see Figure 2). As a function of different temperatures (Figure 8), the presence of free wax at the surface of non-cured MFC film is confirmed by the high intensity of all the wax peaks (2917, 2849, 1473, 1463, 729 and 719 cm^−1^) in the spectrum. Melting and migration of free wax inside the porous MFC film are confirmed by decreasing intensity of the wax peaks with increasing temperatures. At higher temperature like 220 °C, most of the free wax and encapsulated wax present inside the hybrid SMI/wax capsules was released and migrated inside the film, leaving behind thin wax patches of wax over the fibers as shown by the small remaining intensity of the 2917 and 2849 cm^−1^ band (this mechanism was also discussed above in AFM line-profile images). After wax migration at highest temperatures, consequently, the exposure of SMI nanoparticles becomes maximized (as seen in Figure 5) or further imidization of the residual ammonolyzed styrene maleic anhydride happens, resulting in higher intensities of the imide absorption band at 1714 cm^−1^. As a function of different curing times (Figure 9), similar trends are observed with 220 °C. During the first hour of curing, full release of wax was observed, which afterwards decreased due to the migration effect. Latter, due to the complete softening and opening of the capsules, migrated wax show up at the surface resulting in the increase in the surface wax (Figure 6). The effect of curing times for other temperatures on wax migration were also studied (see supplementary information S4), and found to follow the same trend.

**Figure 10:**
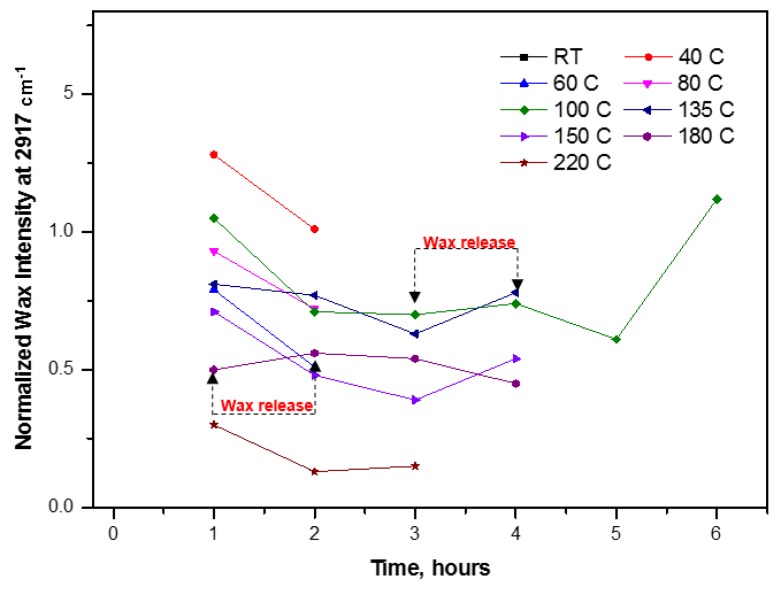
Normalized wax intensity at 2917 cm^−1^ for non-cured (RT) and cured modified MFC films after curing at different temperatures and times.

Moreover, to further quantify the exposure of wax with increasing temperatures and different duration of heat treatments, the normalized wax intensity at 2917 cm^−1^ for all spectra was plotted against the duration of curing for different temperatures (Figure 10). A similar trend for wax release can be seen in all curves. At lower temperatures like 40, 60 and 80 °C, the melting and migration of free wax can be easily followed as a decrease in the wax intensity during 2 h of curing. At intermediate temperatures like 100, 135 and 150 °C, the wax intensity continuously decreased (migration effect) to a minimum value over time, and then a sudden rise is observed after 5, 3 and 3 h, respectively. The sudden increase in wax concentration was probably due to the exposure of the previously migrated under beneath wax, that came in contact after bursting and flattening of hybrid SMI/wax capsules over time (as confirmed by Figure 5 and Figure 6). A very slight increase in the wax intensity can be seen for 100 °C after 3 h of curing up to 4 h, possibly related to a very slow release of encapsulated wax in the capsules at this low temperature. However, it was not possible to detect full release of encapsulated wax for few hours at 135 and 150 °C, as these temperatures remain below the *T_g_* for modified MFC. Despite this, a few capsules burst over increasing curing time (Figure 6) and released wax was migrated at the same time. Both effects balance each other and thus result in an almost constant intensity value for wax in this temperature interval. Finally, at higher temperatures like 180 and 220 °C, full wax release is easily observed after melting of the SMI/wax capsules (Figure 10). The wax that was released after 1 h of curing at 180 °C due to bursting of the capsules, again migrated inside the MFC film over the increasing curing time and explain the further reduction in wax intensity with time. While, at 220 °C, simultaneous release and migration took place during the first hour of curing, because of the much higher temperature than *T_g_* of modified MFC. Further curing after 2 h resulted into the opening and flattering of capsules (Figure 6), leading to the exposure of previously migrated under beneath wax. In conclusion, the higher curing temperatures resulted in the maximum migration of wax and thus left behind very small amounts of wax over the fibers.

#### 2.3.2. Raman Chemical Mapping

The evolution of wax with different temperatures and times was further visualized by dispersive Raman spectroscopy, which was used to create surface chemical maps (1 × 1 mm^2^) shown below. The Raman spectra show characteristic absorption bands for cellulose at (cm^−1^): 3500–3200 (OH stretching); 2896 (CH, CH_2_ stretch); 1464 (C-H-C and C-O-C bending); 1382 (H-C-C, H-C-O, and H-O-C bending); 1150, 1117, 1093 and 898 (cellulose skeletal, C-O-C ring). The major Raman absorption bands for wax are observed at (cm^−1^): 2880 (CH_2_ symmetric stretching) and 2846 (CH symmetric stretching). The main absorption bands for SMI are at (cm^−1^): 1770 (C = O, imide); 1602, 1583, 1184, 1156, 1031 and 1000 (styrene). The Raman absorption bands for cellulose and wax are most significantly recognized in the spectra, along with some minor SMI bands because of the domination by wax at the surface of MFC films.

The Raman chemical maps were created by average intensities of the spectra, and later normalized on the cellulose region. As such, good representation of the wax distribution at the surface of MFC films is obtained, as the highest intensity peaks in the spectra are dominated by wax-related absorption bands. From the applied image processing, the intensity scale (red = highest intensity; green = intermediate intensity; blue = lowest intensity, see on-line version for color figures) related to the amount of presented wax at the surface. The images should be interpreted at a micro-scale level of resolution (different from previous nanoparticle and microfiber scale, as seen in AFM), providing average amounts of chemical moieties over the surfaces.

**Figure 11:**
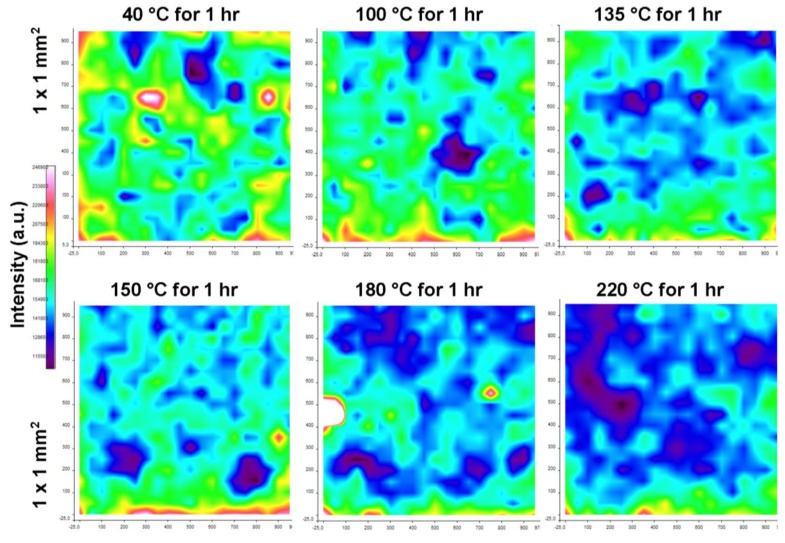
Raman chemical surface mapping for modified MFC films after curing at different temperatures for 1 h (X and Y-axis represent distances 0 to 1000 µm). Same intensity scale bar applies to all images, where, red = highest intensity; green = intermediate intensity; blue = lowest intensity.

The surface maps for MFC films cured at different temperatures for one hour, are shown in Figure 11. As seen by the color intensities, clear variations in the surface chemistry of the films are detected with increasing curing temperature. At lower temperatures, large parts of the surface area are covered with wax and the high intensity values (“red” to “green”) suggest that a large amount of wax is exposed at the surface. The amount of wax is continuously decreasing with increasing temperatures, and finally only thin wax patches (micro-scale) remain distributed over the film surface. These trends are in parallel with the previously discussed intensity variations in FTIR spectra and microscopy, and further confirm quantitatively the proposed mechanisms of progressive wax release and migration.

**Figure 12:**
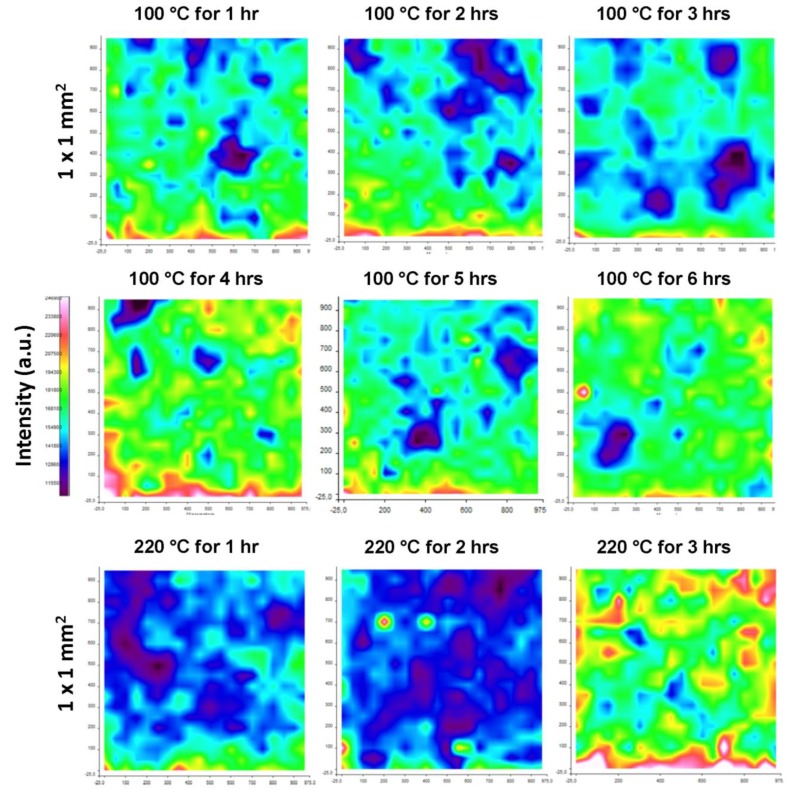
Raman chemical mapping for cured modified MFC films after curing at 100 and 220 °C for different times (X and Y-axis represent distances 0 to 1000 µm). Same intensity scale bar applies to all images, where, red = highest intensity; green = intermediate intensity; blue = lowest intensity.

The effect of curing times on the surface chemistry can be easily visualized by chemical mapping in Figure 12. After curing for only short times at 100 °C, a very slow wax migration was seen with the increasing curing times up to 3 h. Release of encapsulated wax after bursting of the capsules can be seen when cured for 4 h at 100 °C, while there was a further decrease in the surface wax when cured for 5 h due to migration effects. Finally, after 6 hours curing, homogenization and smoothening of the surface with higher concentration of surface wax is observed from the chemical map, as related to the complete opening and softening of the capsules with exposure of under beneath migrated wax (Figure 6). For curing at 220 °C, short times of 2 h resulted in very few wax exposure due to complete melting and migration. The longer curing times at high temperatures cause the spectra to be dominated by imide-related absorption bands, as full imidization of the SMI nanoparticles may happen (see Figure 5, 220 °C magnification). Therefore, the sudden intensity increase at most severe curing condition is related to the homogeneous distribution of imide moieties over the surface.

### 2.4. Physical Analysis

The static water contact angles for non-cured and cured modified MFC films at different temperatures and curing times are presented in Table 1. As a reference, static contact angle of 115° was measured over the pure wax layer deposited over a flat surface. Contact angles for non-cured and cured at 40 °C MFC films were found to be unstable; therefore, the water contact angle was estimated just after 1 s of contact with films. Whereas, the contact angles for all remaining films were stable for at least 60 s (no longer measurements were taken as droplet evaporation than becomes dominating), even though the contact angles were determined after 1 s only (to be comparable with non-cured MFC reference films). The observed trends in relation with previous chemical and morphological film characterization are discussed below.

**Table 1:** Static water contact angle values (°) for non-cured and cured modified MFC films with different temperatures and times.

Time	Curing Temperature (°C)								
**H**	**RT**	**40**	**60**	**80**	**100**	**135**	**150**	**180**	**220**
0	106 ± 8	-	-	-	-	-	-	-	-
1	-	110 ± 6	133 ± 2	130 ± 1	138 ± 1	137 ± 1	137 ± 2	139 ± 1	144 ± 2
2	-	112 ± 8	135 ± 2	133 ± 3	138 ± 3	137 ± 2	139 ± 2	140 ± 2	147 ± 3
3	-	-	-	-	140 ± 1	137 ± 1	145 ± 1	141 ± 1	135 ± 1
4	-	-	-	-	143 ± 1	136 ± 1	137 ± 1	142 ± 1	-
5	-	-	-	-	141 ± 3	-	-	-	-
6	-	-	-	-	137 ± 1	-	-	-	-

RT = Room Temperature, - were not performed.

#### 2.4.1. Contact Angle Evolution with Temperature

**Figure 13:**
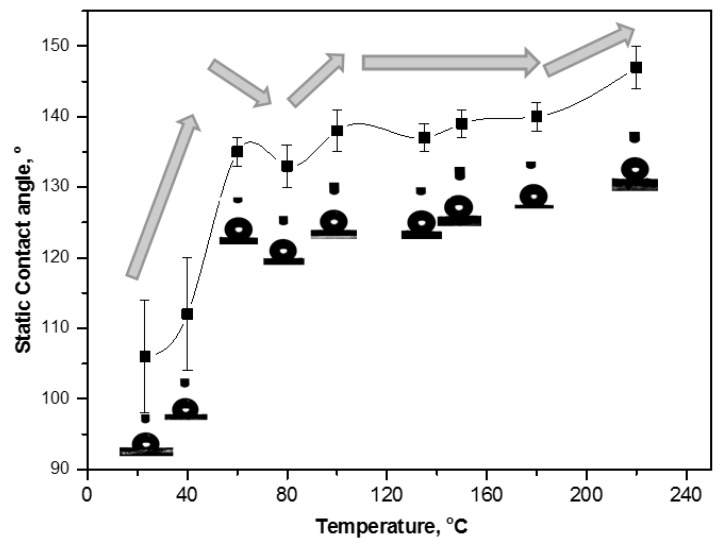
Static water contact angle evolution for non-cured and cured modified MFC films after curing at different temperatures for 2 h.

Figure 13 illustrates the effect of increasing curing temperatures on water contact angles for non-cured and cured MFC films (see Table 1 for exact contact angles values). To understand the progression of hydrophobicity during the whole events, only films that were cured for 2 h were taken into consideration. For non-cured and 40 °C cured MFC films, the contact angles of 106 to 110° were solely dependent on the thick wax granules present on the surface (see Figure 5), comparable to that of a pure wax film. Further heating to 60 °C suddenly increased the contact angle to 135°, as a main result of the partial melting and exposure of surface wax (as determined by DSC, Figure 3). After curing at 80 °C, the contact angle slightly dropped due to complete melting of surface free wax (as determined by DSC, Figure 3). For intermediate temperatures like 100, 135 and 150 °C, a progressive further increase in contact angles and hence hydrophobicity is caused by simultaneous migration of free wax and encapsulated wax (after progressive bursting of SMI/wax capsules) into the porous fiber network (as determined by Figure 10): the latter effects would leave thin wax patches over the highly rough fiber surface which lead to stable and relatively high contact angles. For high temperatures like 180 and 220 °C, the hydrophobicity increased to a maximum with final contact angles of 147°. Bursting of capsules results in the formation of microscale structures (by SEM, Figure 5), having nanoparticles on them by pre-existed dispersion or newly formed from imidization at higher temperatures. It is known that the purely imidized SMI nanoparticle grade coating over paper substrates is conducive to high contact angles at around 102° [20], but the present combination with exposed wax further augments the hydrophobic effect. The above events causing highest hydrophobicity at 220 °C, consequently, created a favorable multiscale micro-to-nano roughness including the microfibrillated fibers (microscale) and SMI nanoparticles (nanoscale), which were covered with a very thin layer of wax (as determined by FTIR, AFM and Raman maps before).

#### 2.4.2. Contact Angle Evolution with Time

**Figure 14:**
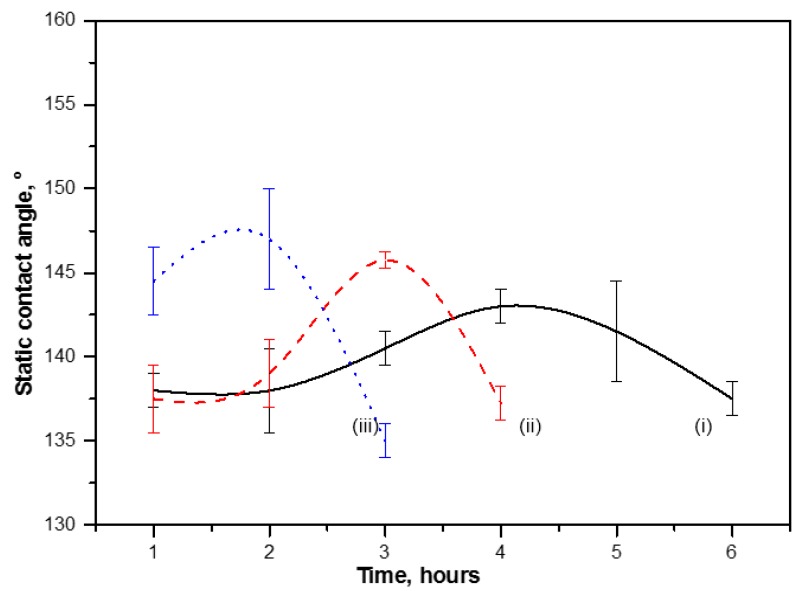
Static water contact angle evolution for cured modified MFC films after curing at (i) 100; (ii) 150 and (iii) 220 °C for different times.

Figure 14 illustrates the influence of curing times on water contact angles for modified MFC films cured at 100, 150 and 220 °C. As discussed above, there has been a considerable effect of curing time on surface morphology and chemistry of films (Figure 6, Figure 10 and Figure 12), and hence influences the contact angles. Water contact angles first tend to increase up to a maximum value at intermediate times and then decrease gradually over longer times. Logically, the curing time for maximized hydrophobicity decreases at higher temperatures. These same trend has been seen (see Table 1 for visualizing the trend of contact angles with increasing temperatures and curing times) for nearly all curing temperatures, except (i) at 135 °C, where the tendency for thermal imidization was much more prominent [20]; and (ii) at 180 °C, which is very near to the *T_g_* of modified MFC and might cause softening. Both effects consequently disturb the wax migration and release. Being a macroscopic parameter, the contact angles are very much sensitive to the combined effects of surface roughness and chemistry, according to the Wenzel model for wetting on hydrophobic surfaces [29]. For present modified MFC films, the surface chemistry is mainly controlled by the presentation of a critical amount of carnauba wax with low surface energy on the outermost film surface. The previous morphological analysis illustrated that the surface irregularities (roughness) of MFC films tend to increase with curing time, while amount of wax at the surface decreases and forms thin wax patches due to the combined effects of release and migration. However, curing for longer times resulted into a surface homogenization and decrease in surface irregularities after exposure to high amounts of previously migrated wax, causing again a drop in the water contact angles.

## 3. Experimental Section

### 3.1. Materials

Microfibrillated cellulosic fibers (Arbocel P1011, Rettenmeyer) were modified by a reaction under aqueous conditions, incorporating hybrid nanoparticles of carnauba wax/styrene-maleimide (SMI), as reported in our previous work [23]. The MFC fibers were charged as a 18% pulp suspension in a 1 L autoclave reactor, along with high-molecular weight styrene maleic anhydride (SMA) copolymer (26 mol% MA, Mw = 80.000 g/mol), ammonium hydroxide, carnauba wax and water, in following amounts: 50 wt% cellulose, 25 wt% SMA, 25 wt% wax and a ratio (ammonium hydroxide)/(maleic anhydride) = 1.01. The reaction was performed with a total solid content of 15%. Under continuous stirring, the reaction mixture was first homogenized for about 2 h at 90 °C under ambient pressure, followed by an increase in temperature up to 160 °C for 4 more hours in parallel with an augmentation of the pressure up to 5.5 bar. The gradual decrease of the initially high viscosity of the reaction mixture over a time interval of 2 to 6 h was an indication for the progressive conversion of the ammonolyzed SMA into a styrene-maleimide (SMI) and formation of a homogeneous pulp suspension of modified MFC fibers including SMI and wax. As a reference experiment, the reaction was repeated under the same conditions with (i) pure SMA; and (ii) a mixture of 30 wt% SMA and 70 wt% wax, forming aqueous dispersions of (i) pure SMI nanoparticles; and (ii) hybrid SMI/wax nanoparticles (used as reference materials for TGA, DSC, DMA and FTIR).

### 3.2. MFC Films Formation and Thermal Curing

The modified pulp suspension was filtered and deposited over a filter paper as thin cast film of 3 cm diameter and later the films were removed from filter paper gently. These films were then pressed between the two plastic sheets to avoid any curling and were freeze dried for 10 h in ALPHA 1-2 LDplus equipment (Christ, Germany) to obtain the film of around 0.8 mm thickness having 480 mg weight. While, the reference materials SMI and SMI/wax were dried under room temperature for longer time. The MFC films were then supplementary heat treated by placing them in a vacuum oven at temperatures of 40, 60, 80, 100, 135, 150, 180 and 220 °C for 1, 2, 3 up to 6 h, respectively.

### 3.3. Characterization

The thermal properties of pure and modified pulp fibers were determined by thermogravimetric analysis (PerkinElmer, Pyris1 TGA), differential scanning calorimetry (PerkinElmer, DSC 8500) and dynamic mechanical analysis (PerkinElmer, DMA 8000). For TGA, sample weights of 5 mg were heated from 30 to 600 °C at the rate of 10 °C/min in a flowing nitrogen atmosphere. For DSC, each sample weight of 5 mg was heated over two cycles from 0 to 250 °C at the rate of 10 °C/min. For DMA, the samples were finely powdered, sieved and filled into material pockets that were heated from 30 to 250 °C at the rate of 2 °C/min, under single cantilever bending mode at the constant frequency of 1 Hz and amplitude of 0.05 mm.

The variation in the morphologies of non-cured and cured modified MFC films were characterized by scanning electron microscopy (SEM), using Hitachi tabletop microscope TM 3000. Although the technique allows researchers to work with uncoated samples due to regulation of the vacuum, better results on MFC films were obtained after deposition of a thin gold coating. The topography of pure and modified pulps at nanoscale was further studied with tapping-mode atomic force microscopy (AFM), using Nanoscope III with a tube scanner from Digital Instruments (Veeco, Santa Barbara, CA, USA) and silicon tips with stiffness k = 50 N/m and resonant frequency of 360 kHz (PPP-NCH, Nanoandmore, Wetzlar, Germany).

The chemical composition of the cured and non-cured MFC films was determined by spectroscopy, using attenuated total reflection Fourier-transform infrared spectroscopy (ATR-FTIR) (Spectrum 65, Perkin Elmer) and dispersive Raman spectroscopy (Raman Flex 400, Perkin Elmer). First, ATR-FTIR was measured on original and cured MFC films at 4000–550 cm^−1^ wavelengths with a resolution of 4 cm^−1^, averaged over 32 scans. Second, the Raman spectra were used to create chemical maps with a surface area of 1 × 1 mm^2^, having 20 × 20 points spaced by a gap of 0.05 mm. A near-infrared 785 nm laser source with 40 mW power at the sample surface was used, recording spectra at a resolution of 2 cm^−1^ between 3200–200 cm^−1^ with exposure time of 2 s for every 10 exposures.

The wettability of the cured films was determined by static contact angles of deionised water measured on a Digidrop equipment (GBX, France) by placing a droplet volume of 4 µL over 60 s and fitting its geometry with a tangent fitting procedure. The measurements were repeated 3 times per sample and reported as average values with statistical standard deviation.

## 4. Conclusions

In this work, a mechanism for actively tuning the wettability of microfibrillated cellulose (MFC) films has been developed in such a way, that a single chemical process is found to be sufficient for the surface modification of the MFC and depending on the required user properties the static water contact angles can be “actively” tuned from 120 to 150° upon selective curing. After synthesis of modified MFC fibers with styrene-maleimide (SMI) nanoparticles and encapsulated plant wax, the contact angles of MFC films could be varied depending on the controlled thermal release of encapsulated plant wax and exposure of imidized nanoparticles that were a priori stored within the nanocomposite film. In parallel with thermal analysis, the modified MFC + SMI/wax nanocomposite seems to form an intimate network structure, with clear transition temperatures related to the release of “free” wax and a glass transition temperature that was only visualized upon dynamic mechanical loading. Consequently, there is a critical temperature range for the release of “free” wax and encapsulated wax within the organic nanoparticles.

The progressive release of encapsulated wax was successfully monitored as a function of temperature and time, by a combination of morphological analysis, infrared spectroscopy and chemical Raman mapping. It can be concluded that the amount of wax presented at the surface depends on balanced mechanisms of progressive release and migration of the wax into the fiber network. As such, a maximum hydrophobicity with the static contact angle of 147° is obtained when cured at 220 °C for 2 h. The latter corresponds to the formation of thin wax patches on top of the MFC fiber structure, benefiting from optimized surface chemistry and (multiscale) morphology over the studied temperature/time interval.

## Supplementary Materials

Supplementary materials can be accessed at: http://www.mdpi.com/1996-1944/7/11/7196/s1.
