# Supplementary material for: Mechanism for Tuning the Hydrophobicity of Microfibrillated Cellulose Films by Controlled Thermal Release of Encapsulated Wax
Source: Materials (Basel). 2014 Oct 28;7(11):7196–216. doi: 10.3390/ma7117196 (PMC5512630; doi:10.3390/ma7117196)

Supplementary Information

Supplementary information S1

Thermal degradation of unmodified and modified MFC, SMI and SMI/wax under flowing oxygen can further increase the rate of degradation, with third degradation peak appeared in the latter stages.

Figure S1. Thermogravimetric analysis (under flowing air) representing weight loss of (i) unmodified MFC, (ii) modified MFC, (iii) pure SMI and (iv) SMI/wax.

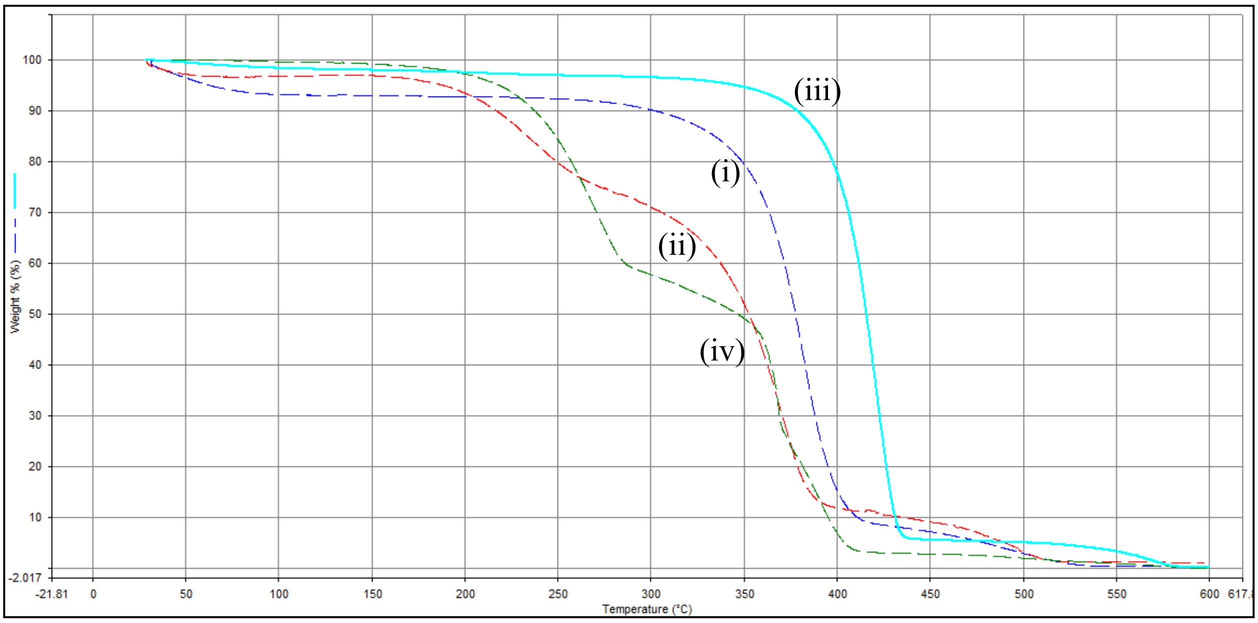

Supplementary information S2

Figure S2 illustrates the complex structure of the walls and inner surface of the SMI/wax capsule, with submicron wall thickness.

Figure S2. Morphology of bursting SMI/wax capsule, (**a**) structure of wall; (**b**) inner surface of capsule.

(a)

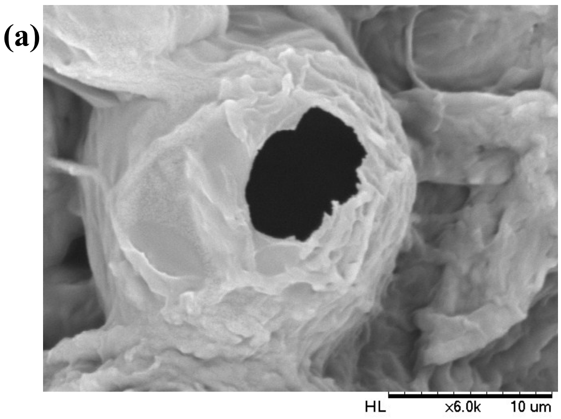

(b)

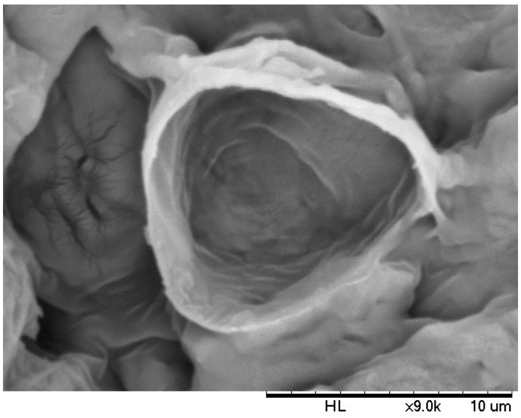

Supplementary information S3

Figure S3 reveals the amount of the deposited wax calculated by line-profile over the single fiber and wax particles. A thin wax patch layer of thickness 15–25 nm was found to be deposited on fibers after curing at 220 °C for 1 h.

Figure S3. Atomic force microscopy of thermally cured modified MFC at 220 °C for 1 h, (**a**) height image (**b**) line-profile over selected regions.

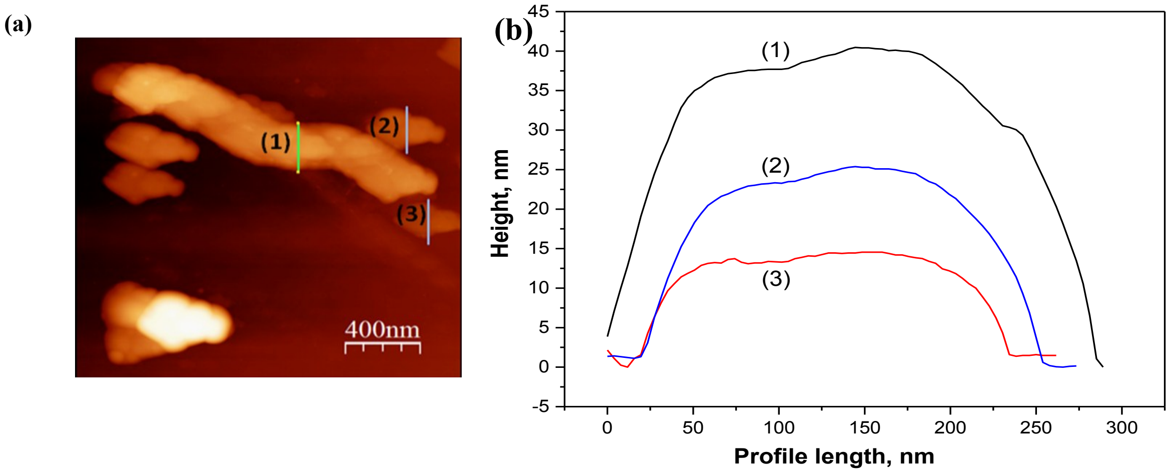

Supplementary information S4

In Figure S4, the effect of curing times for various temperatures on wax migration was studied in detail, and found to follow the same trends as previously described in the paper.

Figure S4. FTIR spectra for different curing times for, (**a**) 40 °C; (**b**) 60 °C; (**c**) 80 °C; (**d**) 100 °C; (**e**) 135 °C; (**f**) 150 °C; (**g**) 180 °C.

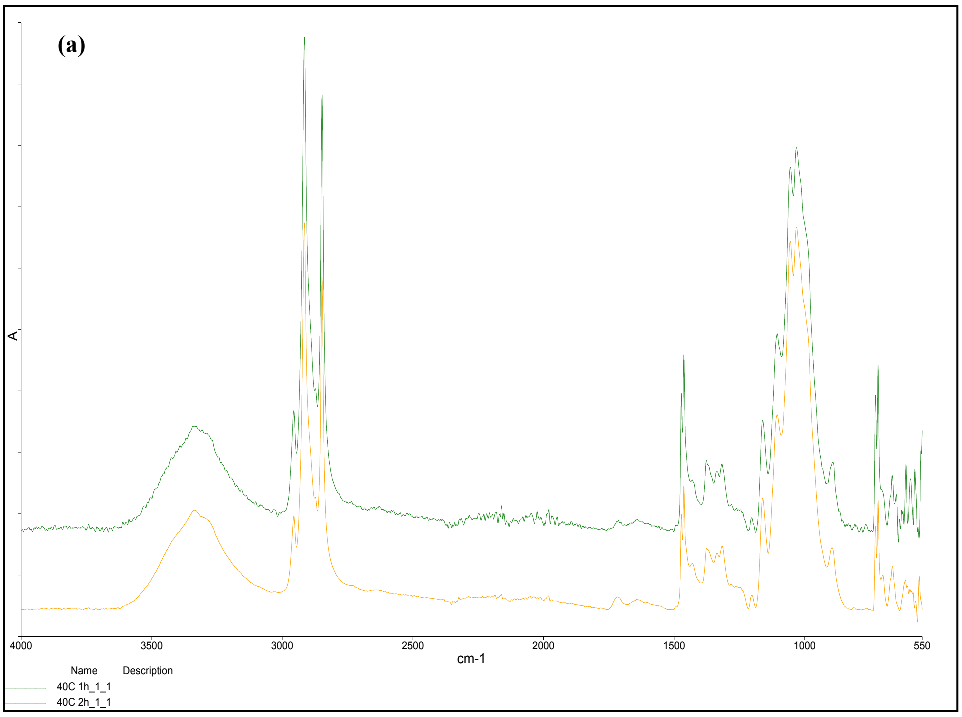

Figure S4. *Cont.*

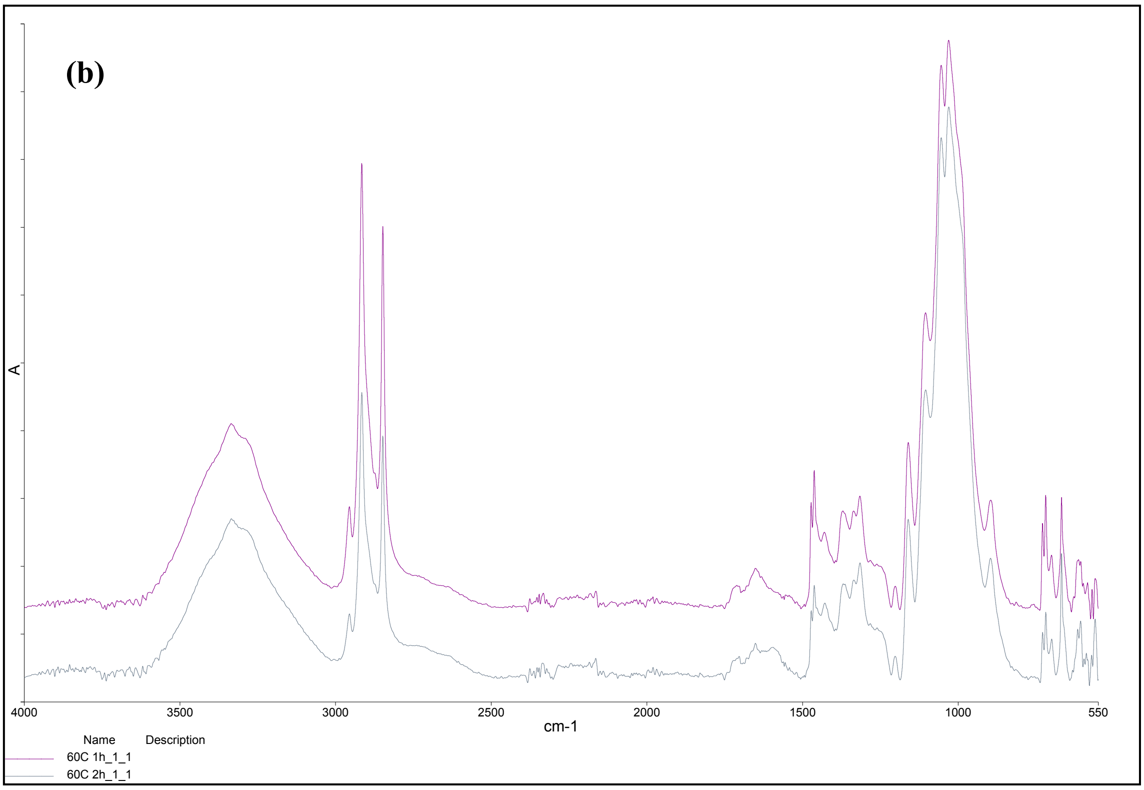

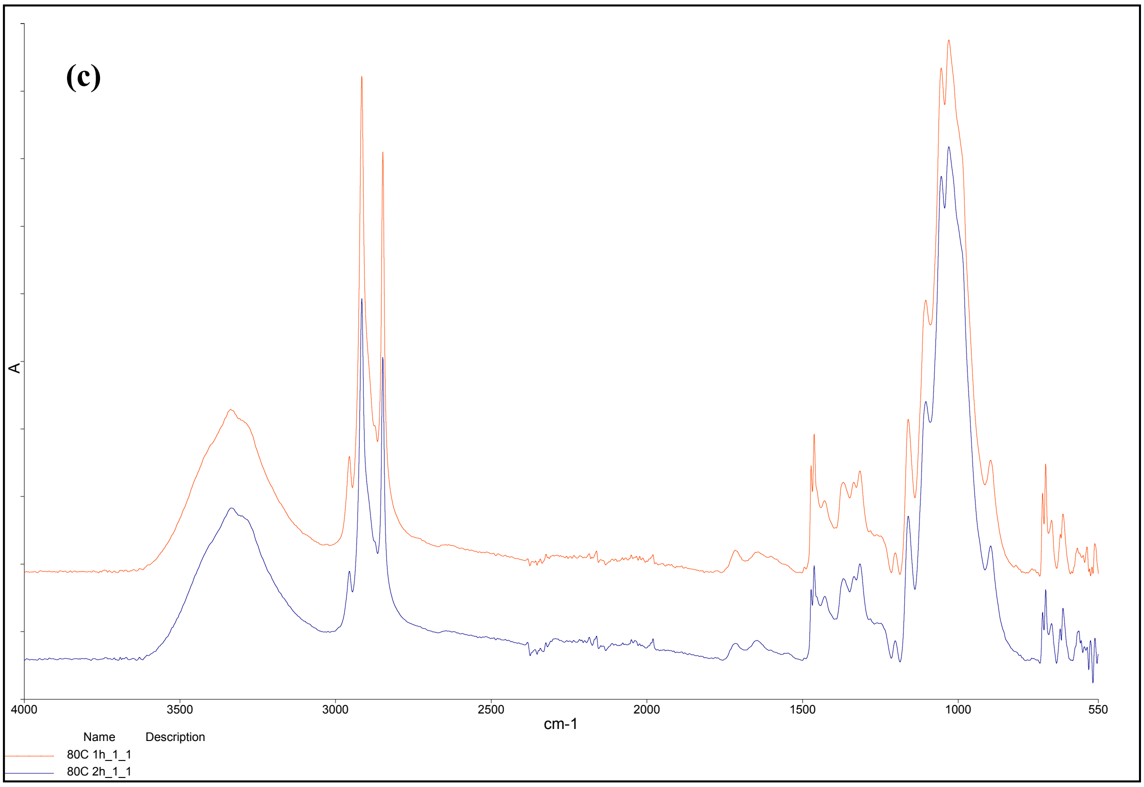

Figure S4. *Cont.*

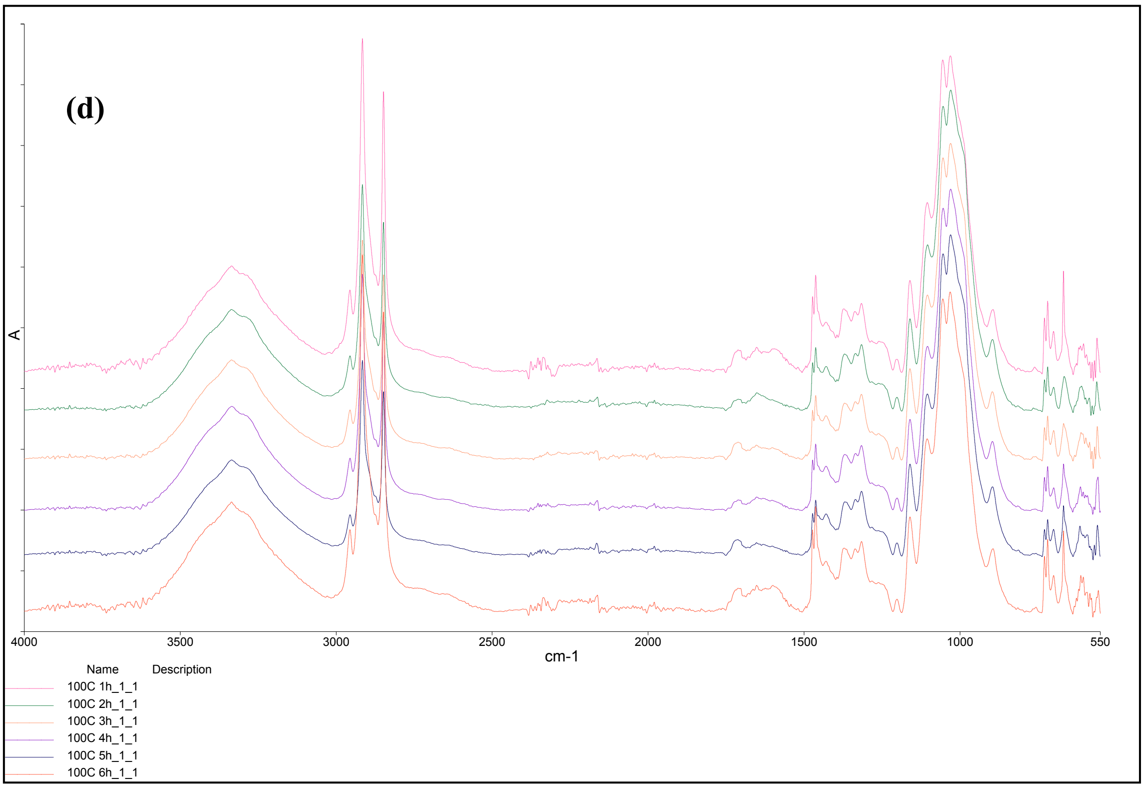

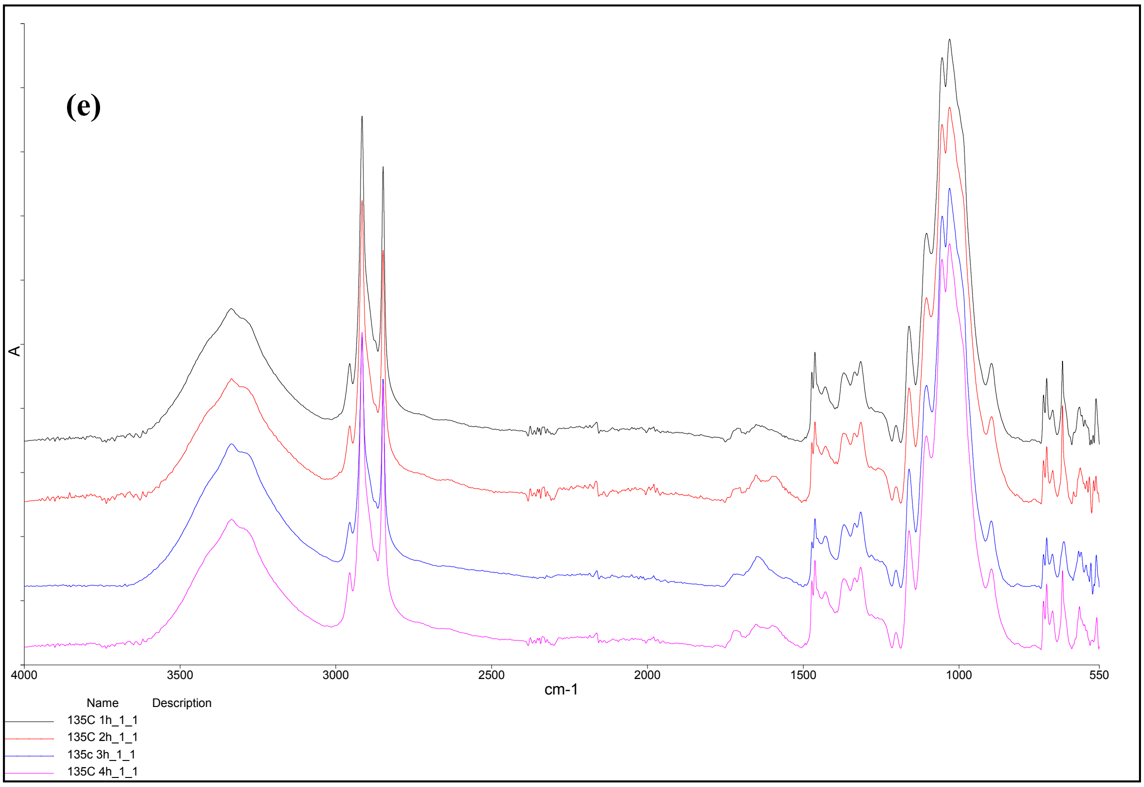

Figure S4. *Cont.*

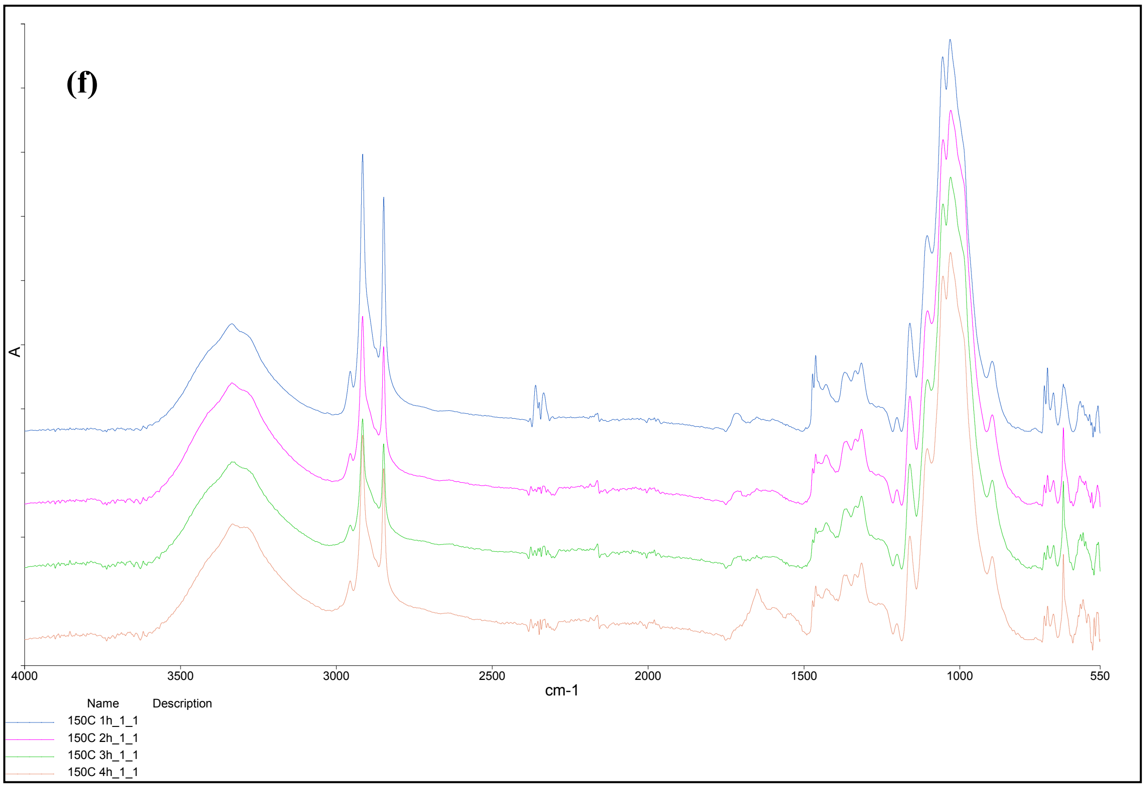

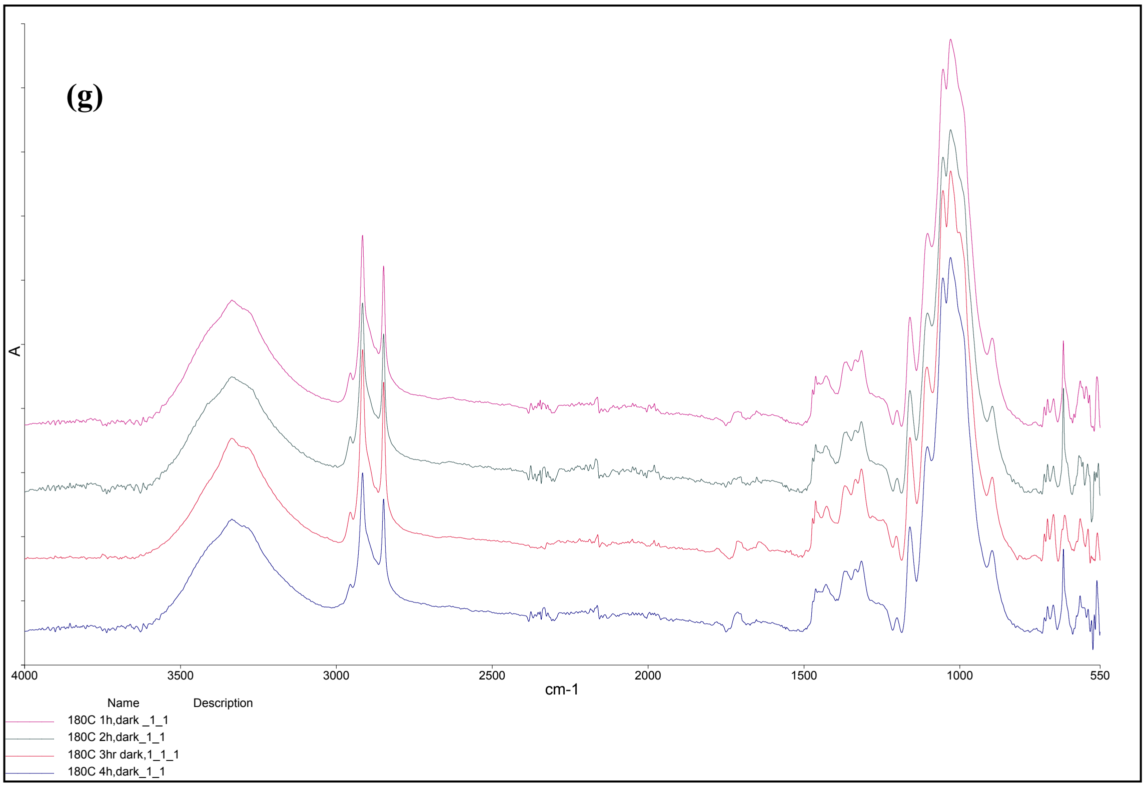

Supplement: Supplementary file 1 [file materials-07-07196-s001.pdf]
